# Xenograft models for pediatric cancer therapies

**DOI:** 10.12703/r/10-11

**Published:** 2021-02-02

**Authors:** Kevin O McNerney, David T Teachey

**Affiliations:** 1 Children’s Hospital of Philadelphia, Divisions of Hematology and Oncology, Philadelphia, PA 19104, USA; 2 Perelman School of Medicine, University of Pennsylvania, Philadelphia, PA 19104, USA

**Keywords:** Xenograft, pediatric cancer, preclinical model, immunotherapy, precision medicine

## Abstract

The prognosis for childhood cancer has improved considerably over the past 50 years. This improvement is attributed to well-designed clinical trials which have incorporated chemotherapy, surgery, and radiation. With an increased understanding of cancer biology and genetics, we have entered an era of precision medicine and immunotherapy that provides potential for improved cure rates. However, preclinical evaluation of these therapies is more nuanced, requiring more robust animal models. Evaluation of targeted treatments requires molecularly defined xenograft models that can capture the diversity within pediatric cancer. The development of novel immunotherapies ideally involves the use of animal models that can accurately recapitulate the human immune response. In this review, we provide an overview of xenograft models for childhood cancers, review successful examples of novel therapies translated from xenograft models to the clinic, and describe the modern tools of xenograft biobanks and humanized xenograft models for the study of immunotherapies.

## Introduction

The prognosis for childhood cancer has improved considerably over the past 50 years. In children, 5-year overall survival (OS) has increased from 58% in 1975–1977 to 84% in 2009–2015; for adults, the increase was from 49 to 69%^1^. Improvements in survival have occurred in most, but not all, types of pediatric cancers. The 5-year OS of childhood acute lymphoblastic leukemia (ALL), for example, has increased from 27 to 91%, and significant improvements have also been seen in retinoblastoma, soft tissue sarcomas, hepatic and germ cell tumors, Wilms tumor (WT), and neuroblastoma^1,2^. These gains can be attributed to well-designed clinical trials that have integrated chemotherapy, radiation, and surgery, as well as improved standard of care, including supportive care. Unfortunately, the prognosis for children with relapsed disease remains poor for most pediatric cancers. For other tumors, including diffuse intrinsic pontine glioma (DIPG) and metastatic solid tumors, survival remains dismal, even in newly diagnosed patients. Additionally, improvements in survival have come with the cost of late effects; approximately 67% of childhood cancer survivors develop at least one chronic health condition^3^.

With the use of histology and immunohistochemistry, pediatric malignancies can be grouped into a relatively small number of subtypes. Early clinical trials were designed based on histopathologic diagnoses. As molecular biology and genetics have advanced, knowledge of the diverse genetic, epigenetic, and proteomic landscape of cancer has ushered in the era of precision medicine and targeted therapies. Additionally, insights in immunobiology and clinical successes of immune-based treatments of select cancers have re-invigorated the field of cancer immunotherapy. The abundance of molecularly targeted and immune therapies in development provide a new horizon for some cancers. However, children are not little adults, and pediatric cancers are biologically distinct from adult cancers. Thus, before new therapies can be moved into the clinic, robust preclinical testing in appropriate models of childhood cancer is needed.

Historically, cell lines and transgenic mice were adequate for the testing of new agents in histopathologically defined disease. While both still have important utility, in an era of precision medicine and immunotherapy, more robust models are needed. Transgenic mouse and cell lines will always remain integral to the study of new agents and disease biology, but models that can reflect the enormous molecular diversity of human cancer are vital.

It is beyond the scope of this review to discuss all the different types of preclinical models for pediatric cancer. Instead, we will provide an overview of the history of xenograft models of childhood cancers, review successful examples of the translation of novel therapies from xenograft models to the clinic, and describe ongoing efforts to establish comprehensive xenograft biobanks and humanized xenograft models for the study of immunotherapies.

## Patient-derived xenografts, molecular characterization, and implications for pediatric cancer treatments

Xenografts involve transplantation of human tumor tissue or cell lines into animals that have been modified to accept the graft^4–7^. Traditional patient-derived xenograft (PDX) models are generated by implantation or injection of human malignant cells into the flank, peritoneum, or tail vein of mice made to be immunodeficient, as mice with intact immune systems will reject the foreign tumor tissue. The human cells may be from fresh tumor samples or from primary cell lines passaged *in vitro* in growth media in monolayer cultures. However, xenografts from established cancer cell lines allow for a limited investigation of different biologies which are often changed by immortalization^8,9^. PDX models were first created with athymic nude mice, then severe combined immunodeficiency (SCID) mice, followed by more immunodeficient models (Table 1)^10–14^. Patient-derived orthotopic xenografts (PDOXs) are generated by transplanting tumor tissue from a patient (often including stroma, fibroblasts, and co-opted immune cells) into a mouse in the same anatomic location as the parent tumor. This strategy preserves the structure of a tumor and helps to more closely recapitulate the tumor microenvironment (TME)^6,15^.

**Table 1.** Common xenograft mouse models used in pediatric cancer research.

Mouse strain	Genetic variant	Mature B cells	Mature T cells	Innate immunity/ NK-cells
BALB/c nu/nu (Athymic Nude)	*Foxn1*	+	-	++
SCID	*Prkdc^scid^*	- (leaky)	- (leaky)	++
NOD/SCID	HLA haplotype H2^G7^/*Prkdc^scid^*	-	-	+
RAG1/2^null^	*Rag1*^null^ and/or *Rag2*^null^	-	-	+
BALB/c/RAG2^null^γ_c_^null^ (DKO)	HLA haplotype H2^d^/*Rag2*^null^/IL-2Rγ_c_^null^	-	-	-
NOD/SCID/Gamma (NSG)	HLA haplotype H2^G7^/*Prkdc^scid^*/*Il2rg^tm1Wjl^*/SzJ	-	-	-
NOD/SCID/γ_c_^null^ (NOG)	HLA haplotype H2^G7^/*Prkdc^scid^*/ *IIl2rg^tm1Sug^/*JicTac	-	-	-
NOD/SCID/β2m^null^	HLA haplotype H2^G7^/*Prkdc^scid^/*β2m^null^	-	-	-
NSG/β2m^null^	HLA haplotype H2^G7^/*Prkdc^scid^*/IL-2Rγ_c_^null^/β2m^null^	-	-	-
NOD/SCID/SGM3 (N/S-SGM3)	HLA haplotype H2^G7^/*Prkdc^scid^*/hSCF/hGM- CSF/hIL-3	-	-	+
Humanized NSG	HLA haplotype H2^G7^/*Prkdc^scid^*/IL-2Rγ_c_^null^*	+ (human)	+ (human)	+/- (human)
Humanized SGM3 (NSG-SGM3 or NSGS)	HLA haplotype H2^G7^/*Prkdc^scid^*/IL-2Rγ^null^/hSCF/ hGM-CSF/hIL-3*	+ (human)	+ (human)	+ (human)
Humanized MI(S)TRG	*Rag2*^null^*Il2Rγc*^null^/hM-CSF/hIL-3/hGM-CSF/ hTPO/SIRPα*	+ (human)	+ (human)	+ (human)

Abbreviations: -, deficient; +, present; ++, robust expression; β2m^null^, major histocompatibility class I beta-2-microglobulin^null^; hGM-CSF, human granulocyte-macrophage-colony stimulating factor; hIL-3, human interleukin-3; hM-CSF, human myeloid-colony stimulating factor; HSC, hematopoietic stem cell; hSCF, human stem cell factor; hTPO, human thrombopoietin; IL-2Rγ_c_^null^, interleukin-2 receptor gamma^null^; NOD, non-obese diabetic; NSG, NOD/SCID/IL-2Rγ^null^; SCID, severe combined immunodeficiency; SGM3, NSG hSCF, hGM-CSF, hIL-3 triple transgenic mice; SIRPα, signal regulatory protein α; RAG, recombination-activating gene. *xenografted with human HSCs

It is important to note that while these models recapitulate pediatric tumors allowing for much-needed investigations, they have limitations. Namely, in order to accept the xenografts, recipient murine immune systems must be abnormal. Furthermore, human tumor biology in living mouse models is altered by the presence of murine cellular and extracellular contaminants. Finally, there is the potential for clonal evolution of the xenograft resulting in a tumor that is different genotypically and phenotypically from the parent tumor tissue. To counteract the latter limitations, these models are validated by comparing the histologic and genetic features of the PDX or PDOX tumor to primary tumor samples. Genomic methods such as whole exome/genome sequencing and transcriptome profiling are used to confirm sentinel genetic alterations that are retained^16,17^. An unfortunate difficulty in xenograft-based research can be access to models. Privately developed xenograft models and xenograft model systems generated by individual institutions may not be as widely accessible. Expanding access to models is an important need for the scientific community.

Both PDX and PDOX models require immunocompromised mice. Conversely, syngeneic mouse models are immunocompetent but are engrafted with tumor lines from the same genetic background to avoid immune rejection. The murine origin of the tumor line limits the applicability of the model to human disease but can be useful in studying the influence of the immune system. Transgenic mice, also known as genetically engineered mouse models (GEMMs), offer another immunocompetent option. These models are generated via the insertion of oncogenes, or knockout of tumor suppressor genes, in murine systems^18,19^. The transgenes can be expressed constitutively or conditionally, leading to endogenous tumor development. Transgenic mice offer the potential of studying genetically defined malignancies in immunocompetent mice, albeit with malignant cells of murine origin.

The advent of “humanized” mice allows examination of the interplay between the human immune system and malignant cells in mouse models. In these models, immunocompromised mice are irradiated, then engrafted with human hematopoietic stem cells derived from human blood, bone marrow, cord blood (CB), fetal liver, and, in some models, fetal thymus for T-cell maturation^20^. These mice can then be xenografted with genetically similar human tumors that will not be rejected. Various modifications to these models have been made to promote human immune system development and prevent xenograft versus host disease (xGVHD), as we will discuss below.

In addition to *in vivo* modeling, recent advances such as the use of three-dimensional (3D) tumor culture systems including tumor explants, tumor-on-a-chip technology, and multicellular tumor spheroids have allowed for *in vitro* study of cancers that more closely approximate the TME. These models can contain numerous cell types including fibroblasts, endothelial cells, and mesenchymal stem cells as well as extracellular matrix and cytokines similar to native human tumors. Additionally, 3D tumor models such as multicellular tumor spheroids have gene expression profiles, gradients of nutrients and oxygen, and biological zones that are more similar to *in vivo* tumors than cells in monolayer cultures, making these valuable options for pediatric cancer modeling^21^.

## Xenograft models in acute lymphoblastic leukemia

One of the earliest successful examples of PDX development is the engraftment of lymphoblasts from patients with ALL into SCID mice. SCID mice lack mature B- or T-cells and are capable of accepting ALL blasts at a rate of ~15%^22–25^. Interestingly, the low engraftment rates had prognostic utility in one study, as blasts from patients with higher risk, poorer prognosis ALL were more likely to engraft^24^. As only ALL blasts from high-risk disease engrafted in SCID mice, only a genetically narrow population was studied using early models. In order to study a more comprehensive subset of patient-derived ALL, more immunodeficient mouse models were required. The backcrossing of SCID mice with non-obese diabetic (NOD) mice led to a more completely immunodeficient NOD/SCID mouse, which accepts ALL patient samples at rates of ~75%^26–28^. Although, in NOD/SCID mice, persistent natural killer (NK)-cells continue to mediate leukemia rejection. On the NOD/SCID background, interleukin-2 receptor gamma chain (*IL2Rγ*) was modified to further impair the innate immunity of these animals^29^. The resultant NOD/SCID/gamma (NSG) mice have no mature B-cell, T-cell, or NK-cell immunity to mediate rejection and therefore have higher rates of ALL engraftment^29^. Knockout of the major histocompatibility complex class I beta2-microglobulin (*β2m*) in NSG mice allows engraftment of >90% of ALL patient samples^30^.

Pediatric ALL xenografts have been used extensively to study disease biology by expanding the number of blasts for mechanistic evaluation. A few hundred thousand blasts injected into a mouse can expand to billions of blasts for *ex vivo* analysis. ALL xenografts have also been used to test novel therapies (Table 2). Blinatumomab, a bi-specific T-cell engager (BiTE) for CD19 and CD3, was investigated in NOD/SCID mice supplemented with human T-cells, providing efficacy and safety data for clinical trials^31–34^. Other agents broadly tested in ALL models that have led to clinical trials include immunotherapies such as chimeric antigen receptor (CAR) T-cells and monoclonal antibodies targeting CD19 and CD22 (discussed below), small molecule inhibitors targeting NOTCH, CDK4/6, PI3K/Akt/mTOR, JAK/STAT, and mitogen-activated protein kinase (MAPK), and cytotoxic chemotherapeutics^32,45,47–49,93,94^. ALL xenograft models have been used to compare the differential sensitivity of drugs in diagnostic:relapse pairs generated from individual patients to study clonal evolution^50^. Finally, ALL xenografts have been used to study targeted therapies in high-risk ALL subtypes, including BCL2 inhibitors in hypodiploid ALL, JAK/STAT inhibitors in early T-cell precursor (ETP) ALL, and ABL-kinase inhibitors in Ph-like ALL^49,95,96^.

**Table 2.** Examples of experimental therapies used in pediatric cancer xenograft models.

Pediatric cancer	FDA-approved agent tested	Investigational agent tested	Mouse strain
ALL	Venetoclax^35–37^ CAR T-cells^38–43^ Imatinib^44^ Blinatumomab^34,45^	Denintuzumab^46^ Palbociclib^47^ γ-secretase inhibitor^48^ Ruxolitonib^49^ Trametinib^50^ MI-3454^51^ Navitoclax^52^	NSG^49–52^ C3H2^38^ MI(S)TRG^53^ B6.SJL CD45.1+ Ccnd2^–/–^ and Ccnd3^–/–^^47^ NOD/SCID^49,52^
AML	Venetoclax^35–37^ Gemtuzumab^54^	Cobimetinib^55^ MI-3454^51^ Quizartinib^56^ VTP50469^57^	NSGS^55^ NOD/SCID^56^ NSG^35,51,57^
CML	Dasatinib^58,59^ Nilotinib^60^ Imatinib^44^	GSK343^58^ Hydroxychloroquine^61^	DBA/2J^60^ NSG^58,61^
Hodgkin lymphoma	Brentuximab^62–64^ Nivolumab^65,66^	Ruxolitinib^67,68^ Navitoclax^64^	NOD/SCID^64^ NSG^67,68^
NHL	Rituximab^69^ CAR T-cells (DLBCL)	Obinotuzumab^70^ Ofatumumab^70^ Fedratinib Ruxolitonib^67,68^ Midostaurin^71,72^	SCID^67,68,70,71^ NSG^72^
Neuroblastoma	Dinutuximab^73^	Galunisertib^74^ CAR T-cells^75^ CAR NKT-cells^76^ Crizotinib^2,77–79^* Ceritinib^77^ CGM097^78,80^ Lorlatinib^81^	SCID^77,78^ NSG^74,82^ Hu-NSG^76^ BALB/C-Nu^78,81^
Wilms tumor		Lorvotuzumab– mertansine^83^ WT1-directed vaccine^84^ AZD1775^85^	NOD/SCID^83,85^ BALB/c^84^
Rhabdomyosarcoma		Panobinostat, bortezomib, AZD1775^86^	NOD/SCID^86^ NSG^86^
Ewing sarcoma		Mithramycin^87^ Trabectidin^88^	SCID^87^ Crl-Nu/Fox1- Nu^88^
Osteosarcoma		ML264^89^	BALB/C-Nu^89^
Medulloblastoma		Vismodegib^19,90,91^	Ptch1^+/–^ & Tp53^–/–^^91^ NSG^90^
Glioblastoma		Erlotinib^90^ Veliparib^92^	NSG^90^ SCID & BALB/c Nu^92^

Abbreviations: ALL, acute lymphoblastic leukemia; AML, acute myeloid leukemia; C3H, C3H/HeN-MTV-negative; CML, chronic myeloid leukemia; DLBCL, diffuse large B cell lymphoma; hu-NSG, humanized NSG; NKT, natural killer T; NHL, non-Hodgkin lymphoma; NOD, non-obese diabetic; NSG, NOD/SCID/IL-2Rγ^null^; SCID, severe combined immunodeficiency. *FDA approved in non-small cell lung cancer

## Xenograft models in acute myeloid leukemia

In contrast to ALL, acute myeloid leukemia (AML) xenografts have been more difficult to establish in NOD/SCID and NSG mice because of low levels of engraftment and poor proliferation of malignant cells^97–99^. Other model systems have more robust engraftment, including NOD/SCID/β2m^null^ mice and NOD/SCID mice with transgenic expression of stem cell factor (SCF), granulocyte/macrophage-colony stimulating factor (GM-CSF), and interleukin-3 (IL-3) (N/S-SGM3 mice), which facilitate AML engraftment and proliferation^100^. AML PDX models have been used to investigate small molecule inhibitors such as the BCL-2 inhibitor venetoclax, which led to clinical trials in adults and children and eventual FDA approval in adults >75 years of age^35–37,101,102^. AML PDX models have been used to investigate combination therapies such as venetoclax with the MAPK inhibitor cobimetinib in NSG mice modified with SGM3 transgenes (NSGS mice)^55^. This work has also led to clinical trials. Similar to ALL models, AML PDX models have been used to study targeted therapies, including FLT3 inhibitors in FLT3/ITD AML PDX models and menin inhibitors in KMT2A-rearrangement (KMT2A-r) AML^51,56,57^. Immunotherapies have also been used in AML PDX models (discussed below).

## Xenograft models in chronic myeloid leukemia

Chronic myeloid leukemia (CML) is a proliferative disorder involving the accumulation of early myeloid precursors as a result of the formation of the *BCR-ABL1* fusion protein formed by reciprocal translocation of chromosomes 9 and 22^103^. CML can have three phases as blasts acquire additional genetic alterations: chronic phase, accelerated phase, and blast crisis. In early CML xenograft experiments that used SCID mice, only PDXs from patients in blast crisis could reliably engraft^104^. Later, irradiated SCID mice and NOD/SCID mice accepted PDXs from patients in chronic phase as well as blast crisis^105,106^. Another study found that CML patient samples with a high percentage of long-term culture-initiating cells (LT-CICs) were most capable of consistent long-term engraftment in irradiated NOD/SCID and NOD/SCID/β2m^null ^mice^107^. Human CB CD34^+^ cells have been transduced for *BCR-ABL1* expression with resultant leukemia initiation in NOD/SCID mice. Engraftment was further improved when *BCR-ABL1*-expressing human cells were made to co-express BMI1, suggesting that BMI1 may be a valuable target in CML therapy^108^.

One of the main hurdles in CML therapy is tyrosine kinase inhibitor (TKI) resistance. NSG xenograft models using TKI-resistant CML lines and PDXs have been used to explore mechanisms of TKI resistance^58,60,61^. In these studies, autophagy was found to contribute to TKI resistance, and blockade of autophagosome formation with hydroxychloroquine was shown to have synergistic action with mTOR inhibitors, prompting interest in mTOR inhibitor/autophagosome inhibitor combination therapy for patients with TKI resistance^61^. Albeit, similar results were not found in the clinic^44^. CML PDX models have been used to test numerous second- and third-generation TKIs as well as combination therapies such as EZH2 inhibitors in combination with TKIs^58,109,110^.

## Xenograft models in Hodgkin lymphoma

Hodgkin lymphoma (HL) xenografts were initially challenging to generate owing to the relative scarcity and poor growth of Hodgkin and Reed-Sternberg (HRS) cells, the malignant cells in HL. At least one group successfully generated primary xenografts from primary HL tissue in SCID mice, but only with engraftment from 3/13 patients (23%), and with high rates of Epstein-Barr positivity in non-HRS cells (80–100%). Furthermore, the tumors generated from these engraftments had three different morphologic patterns: lymphoproliferative disease, anaplastic large cell lymphoma (ALCL), and Hodgkin-like^111^. In SCID mice, xenografts from HL cell lines generated tumors at a rate of 57%^112,113^. Because of the difficulty of primary tumor engraftment, it is now more common for HL xenograft experiments to utilize cell lines instead of primary patient samples. HL cell line xenograft models in SCID mice have been used to test novel therapies, including monoclonal antibodies targeting CD30, such as AC10 and 5F11^114–116^. 5F11, however, was not effective in clinical trials^117^. In contrast, cell line HL xenograft models also were used to test the efficacy of brentuximab, which was derived from cAC10 conjugated to monomethyl auristatin E via a valine-citrulline peptide linker^118^. Brentuximab demonstrated both preclinical and clinical efficacy and is now FDA approved^62,63,119^.

## Xenograft models in non-Hodgkin lymphoma

Non-Hodgkin lymphoma (NHL) mouse models have also been generated and provided valuable preclinical data that have led to progress in the clinical domain. For example, cells from patient-derived ALCL tumors were injected into the flanks of NOD.cg-Prkdc^scid^ IL2rg^tm1Sug ^(NOG) mice with successful engraftment and lymphoma dissemination^120^. Brentuximab, mentioned above, also had demonstrated efficacy preclinically in ALCL xenografts into SCID mice, leading to translation with efficacy in patients^62,118^. Primary mediastinal large B cell lymphoma (PMBL) is a subtype of diffuse large B-cell lymphoma (DLBCL) that occurs predominantly in young adults^67^. PMBL xenograft models have not been generated with primary tumor samples but have been generated using Karpas1106P and MedB1 cell lines, allowing treatment responsiveness assessment with targeted JAK2 inhibition with fedratinib and ruxolitinib as well as with the anti-79b antibody–drug conjugate polatuzumab vedotin^67,68,121^. Interestingly, PMBL cell lines were shown to have sensitivity to ruxolitinib in both *in vitro* and Karpas1106P-xenografted NSG mice, although this did not translate to therapeutic responses in a small phase 2 trial^67,68^. A phase 1 clinical trial evaluating polatuzumab in patients with PMBL is now recruiting (NCT04231877).

Burkitt lymphoma (BL), a NHL representing 40–50% of all pediatric lymphomas, was first studied in transgenic mouse models (*MYC* gene under the control of IgH or IgL sequence)^122–124^. Subsequently, PDX BL models were generated from affected patient lymph nodes in athymic nude and SCID mice, demonstrating metastatic potential in SCID mice, but not in athymic nude mice^125^. These models have been used to study novel agents such as the pan-protein kinase C (PKC) inhibitor midostaurin with and without rituximab^71,126^. Promising results in preclinical models have led to ongoing clinical trials^71^.

## Xenografts in neuroblastoma

Solid tumor xenograft models have proven useful for the study and development of effective treatment regimens in pediatric cancers. In neuroblastoma, orthotopic and systemic xenograft models were developed in BALB/c/Rag2^null^γ_c_^null^ mice with adrenal or intravenous injection of human IMR-32 and IGR-N91 neuroblastoma cell lines^127^. In NSG mice, patient-derived primary neuroblastoma samples that were cryopreserved after surgery were used to create PDOX models that demonstrated invasive growth patterns and retention of patient-specific genetic markers, as well as the ability for the tumors to be monitored with PET and MRI imaging^128^. These models were used to test dinutuximab (Unituxin^TM^), an IgG1 human/mouse chimeric switch variant of 14G2a, targeting GD2 on neuroblasts^73,129,130^. Promising preclinical results led to clinical trials and eventual FDA approval of dinutuximab in children with high-risk neuroblastoma with at least partial response to first-line multi-agent, multi-modal therapy^73^. More recent preclinical studies have tried to improve the efficacy of dinutuximab by enhancing the immune response. These have included the study of the TGF-β inhibitor galunisertib as well as the use of activated NK-cell infusion with dinutuximab in neuroblastoma-xenografted NSG mice; both of the combinations were superior to monotherapy^74,131^.

Neuroblastoma xenografts have also been used to test targeted therapies. As an example, neuroblastoma cell lines with ALK variants or ALK amplification in athymic nude and SCID mice have been used to study ALK inhibitors alone, and in combination with chemotherapy, demonstrating improved tumor control and prolonged survival in these models^77,99,132,133^.

## Xenografts in Wilms tumor

Initial attempts to make PDXs from WT had engraftment rates of approximately 30%, and cell lines made from WT samples would not engraft^134^. Then, using 1×3×3 mm^3^ minced WT fragments implanted underneath the renal capsules of athymic nude mice, one group demonstrated a 67% engraftment rate^135^. When another group xenografted minced WT tumors in NOD/SCID mice, engraftment rates of 80% were found^83^. This was attributed to the proportion of the WT sample that included the blastemal component, which was found to have a distinct gene expression pattern and was thought to confer stem-like properties to the xenografts^83^. Finally, another group generated a WT PDX library, successfully xenografting 45 WT patient samples into SCID mice. These PDXs demonstrated consistent enrichment of the blastema relative to primary tumors^136^. This PDX library was treated with doxorubicin, actinomycin D, and vincristine, demonstrating sensitivity in xenografts derived from favorable histology WT, and more frequent refractoriness in PDXs from anaplastic WT samples^136^. WT models have also been used to test novel therapies, including a human NCAM antibody–drug conjugate called lorvotuzumab–mertansine. WT tumors were completely eradicated in the mice; however, the drug was not active in a phase 2 clinical trial^83^.

## Xenograft models in sarcomas

Sarcomas including rhabdomyosarcoma, Ewing sarcoma, osteosarcoma, rhabdomyosarcoma, desmoplastic small round-cell tumors, high-grade sarcomas, retinoblastoma, adrenocortical carcinomas, and rare solid tumors have also had xenograft models generated successfully. To improve understanding of the complexity of these pediatric solid tumors and identify drug vulnerabilities, one group generated PDOX models in athymic nude and NSG mice using samples from 168 patients with an overall 45% engraftment rate^86^. In this large effort, they established 67 PDOXs in 12 solid tumor types and performed molecular characterization using immunohistochemistry (IHC), transmission electron microscopy (TEM), genetic sequencing, epigenetic analysis, and tumor clonal analysis over time^86^. Patient-derived tumors were dissociated and cultured for *in vitro* drug sensitivity screening, which identified that HDAC and proteasome inhibitors had activity across multiple tumor types and cell lines and that a WEE1 inhibitor called AZD1775 was particularly active in rhabdomyosarcoma cells. Rhabdomyosarcoma PDOXs were then treated with the HDAC inhibitor panobinostat in combination with bortezomib, albeit without response^86^. In contrast, AZD1775 combined with irinotecan and vincristine was more effective than irinotecan and vincristine alone in rhabdomyosarcoma PDOX, leading to an ongoing early phase trial^86^.

In Ewing sarcoma, malignant cells depend upon activity of the EWS-FLI1 transcription factor, making it an attractive target for study. In preclinical models, effective inhibition of this transcription factor’s activity required high concentrations of EWS-FLI1 inhibitors^88,137^. In a phase 1/2 clinical trial of the EWS-FLI1 inhibitor mithramycin, comparable concentrations could not be achieved because of hepatoxicty^138^. More recent studies using Ewing’s sarcoma xenografts in nude mice have demonstrated differentiation of Ewing sarcoma tumors with the use of another EWS-FLI1 inhibitor, trabectedin, in combination with irinotecan, prompting interest in this combination in clinical trials^88^.

## Xenograft models in central nervous system malignancies

Central nervous system (CNS) malignancies have been particularly difficult to study in preclinical models. Nevertheless, PDX and PDOX models have been generated for multiple CNS malignancies. In general, PDOX models with intracranial tumor placement have the advantage of recapitulating the blood–brain barrier but the difficulty of monitoring disease progression over time. PDX flank models can be monitored more easily but do not have the same blood–brain barrier seen in parent tumors, which weakens the applicability of the model. Intra-orbital CNS tumor xenografts allow for engraftment in an immune-privileged site that can be monitored but with small tumor volumes^139^.

In medulloblastoma, the most common CNS tumor in children, patient-derived samples have been successfully xenografted into the flanks of nude mice and could be serially passaged *in vivo*^140,141^. These studies have allowed for the study of medulloblastoma tumor biology and molecular characterization, leading to a greater understanding of the disease^141,142^. PDX and PDOX mouse models now exist for all four major molecular groups of medulloblastoma^19^. In one study, medulloblastoma samples with *SHH* aberrations were treated with the smoothened inhibitor vismodegib and had prolonged survival compared to a vehicle-treated control^90^. For high-grade gliomas, mice have also been xenografted with patient samples for preclinical investigation of novel therapies. For example, high-grade gliomas with *EGFR* amplification were xenografted and treated with the EGFR inhibitor erlotinib, demonstrating improved survival relative to control^90^. Vismodegib and erlotinib remain under clinical investigation in pediatric patients with medulloblastoma and high-grade gliomas (NCT01878617 and NCT00602667).

Xenograft models of glioblastoma multiforme (GBM) and other high-grade gliomas have also been developed^143,144^. Orthotopic and flank injection of patient-derived glioblastoma stem cells were used to show that the stem cells are capable of differentiating into endothelial cells, providing a source of vascularization for the highly aggressive tumor. This endothelial differentiation was therefore postulated as a therapeutic target to prevent tumor angiogenesis in the treatment of GBM^145^. GBM models have also been used for preclinical testing of a number of novel therapies, including STAT3 inhibitors, NAMPT inhibitors, radiation + checkpoint inhibitors, EGFR inhibitors, and PARP inhibitors^146–151^.

## Xenograft consortia and targeted treatments

A number of groups have developed robust consortia of PDX models that allow for the investigation of disease biology through molecular characterization, drug screening, and high-throughput testing of therapies^16,86,90^. One such example is the pediatric preclinical testing consortium (PPTC), which has generated over 390 patient-derived tumor xenografts that have been molecularly characterized by single nucleotide polymorphism (SNP) analysis, comparative genomic hybridization (CGH), whole exome/whole transcriptome analysis, and short tandem repeat testing^16,152,153^. Other large xenograft consortia include the Childhood Solid Tumor Network, Children’s Oncology Group Cell Culture and Xenograft Repository, Targeting of Resistance in Pediatric Oncology Program, IMI2 ITCC-P4, and the European PDX Consortium.

One group generated a PDX biobank of drug-resistant ALL samples from 60 patients with high-risk leukemia^72^. These leukemias were characterized extensively with genomic profiling to ensure the engrafted tumors maintained the genetic alterations found in the primary patient samples. Venetoclax was shown to be highly active in KMT2A-r ALL and TCF3-HLF ALL *in vitro.* Dasatinib and venetoclax were demonstrated to have a high level of activity against a group of T-ALL samples *in vitro*. The *in vitro* activity was confirmed in PDX models in NSG mice, and, as a result, a patient with refractory T-ALL was treated with dasatinib and achieved a 5-month remission^72^. The PPTC has also reported prediction of clinical efficacy with its drug screening and PDX models^52^. The molecular profiling, drug sensitivity screening, and *in vivo* xenograft testing that these consortia provide allows for powerful and predictive modeling for molecularly defined malignancies.

Preclinical PDX biobanks can help to guide the determination of which drugs should have the highest priority for introduction into clinical trials for specific subsets of patients. There are several cohorts running clinical trials that first genetically profile tumor samples and then enroll subjects on treatment arms based on their profiling results and existing preclinical and/or clinical efficacy data. The NCI-COG Pediatric MATCH is a large phase 2 trial that provides targeted therapies for relapsed/refractory pediatric solid tumors, NHL, and histiocytic disorders on the basis of patient tumor genetic features. This study currently has 10 treatment arms and has a match rate of 24%, which is much higher than the predicted 10%^154^. Other similar programs include the MAPPYACTS, SMPaeds, iTHER, INFORM, and TARGET trials^155^. Molecularly targeted clinical trials are expected to grow as molecular targets and drugs continue to be developed through *in vitro* tumor characterization and PDX modeling.

## Xenograft models for study of immunotherapies

Cancer immunotherapies have demonstrated substantial efficacy for certain malignancies that have been refractory to traditional treatment modalities and have enormous potential for improving outcomes in others. However, many types of cancer have been immunotherapy refractory, prompting active preclinical research in xenograft models. The study of immunotherapies in these models presents a unique challenge in that their efficacy and toxicities are typically most accurately modeled in animals with intact immune systems.

### Monoclonal antibodies

A classic example demonstrating the need for an intact immune system for the preclinical study of immunotherapies was an experiment evaluating the efficacy of the anti-CD20 monoclonal antibody (moAb) rituximab in SCID mice xenografted with CD20^+^ Raji lymphoma cells, with or without depletion of NK-cells and neutrophils. NK-cell and neutrophil-replete mice that were treated with rituximab had greater survival when compared with NK-cell or neutrophil-depleted mice treated with rituximab and NK-cell/neutrophil-replete mice treated with placebo, illustrating the importance of neutrophils and NK-cells for antibody-dependent cellular cytotoxicity (ADCC)^156^. Another study evaluated the role of Fcγ receptors in the efficacy of the anti-HER2 moAbs trastuzumab and rituximab in breast cancer- or lymphoma-xenografted mice, respectively. BALB/c nude mice were modified to be deficient in the activation Fcγ receptors FcγRI and FcγRIII or the inhibitory receptor FcγRIIB. These mice and wild-type BALB/c nude mice were xenografted with HER2^+^ breast cancer cells or CD20^+^ Raji lymphoma cells. They found that the greatest protection offered by the moAbs was in mice that were deficient in FcγRIIB but with intact FcγRI and FcγRIII. No protection was offered by moAbs in FcγRI- and FcγRIII-deficient mice^69^. SCID mice and nude mice provided good models for the study of these therapies because of their largely intact innate immune systems, which mediate the ADCC response. However, these mice do not completely recapitulate an intact immune system, and their innate immunity is murine in origin.

### Chimeric antigen receptor technology

CAR T-cells directed against CD19 were initially studied in syngeneic mouse models with murine-derived lymphoma cells and murine CAR T-cells. Syngeneic models have the advantage of being immunocompetent, with the disadvantages of diminished murine CAR T-cell persistence (when compared with human CAR T-cells) and the use of murine instead of human tissues^38,157^. Illustrating this, in one syngeneic model, BALB/c mice xenografted with murine lymphoma cells did show cytokine elevation (IFN-γ and TNF-α) when treated with murine CAR T-cells, suggesting systemic inflammation secondary to CAR T-cell treatment. However, this did not match the cytokine release pattern later seen in patients with cytokine release syndrome (CRS), one of the main toxicities associated with CAR T-cells^158^. Xenograft experiments testing CAR T-cells against B-ALL are now most often carried out in NSG mice, allowing study of the human CAR T-cells against human leukemia^157^. In these models, CD19-directed CAR T-cells demonstrated profound anti-leukemia efficacy, but, owing to the immunodeficiency in these mice, CRS was not observed^157^.

The successes seen with CD19-directed CAR T-cells in preclinical models translated into robust clinical responses in relapsed/refractory pediatric B-ALL patients treated with the CD19-directed CAR T-cell product tisagenlecleucel (Kymriah)^39,40^. Based on these results, tisagenlecleucel became the first FDA-approved cell-based gene therapy in 2017. Two major toxicities experienced by CAR T-cell recipients are CRS, a syndrome characterized by severe systemic inflammation with elevations in IL-6 and IFN-γ that occurs in the vast majority of patients treated, and neurotoxicity, which can range in severity from seizures to death^40^. CRS and neurotoxicity were not observed in early models because of lack of human immune cells and lack of murine tissue responsiveness to human cytokines^40,41^.

Now, improved preclinical models have been developed that allow for the prediction of immune-mediated toxicities of CAR T-cells and serve as excellent tools for ongoing research in this area. One example is the use of humanized NSG mice made transgenic for human cytokine production to promote human immune system development (NSGS or NSG-SGM3 mice). Humanized NSGS mice were xenografted with patient-derived ALL, then treated with CAR T-cells derived from the humanized lymphocytes^41^. Interestingly, the treated mice demonstrated neurotoxicity and CRS differentially mediated by IL-1 and IL-6, while the same effects had not been shown in NSG mice without the transgenic cytokine expression^41^. Furthermore, human monocytes and macrophages were found to be the main source of these cytokines, explaining why previous immunodeficient CAR T-cell-treated xenograft and syngeneic models may not have experienced these toxicities^41^. Finally, an IL-1-directed antibody, anakinra, was used to treat mice with neurotoxicity, reducing its severity and lethality^41^. Such a model will likely help to predict toxicities in future studies of CAR T-cell therapies.

Humanized NSG mice have also been used to generate ALL and CAR T-cells from the same human tissues (autologous CAR T-cells), as is typically done in the clinic. With one method, irradiated NSG mice were xenografted with human fetal thymus and CD34^+^ fetal liver cells (FLCs)^159^. CD34^+^ FLCs were also transduced with a retrovirus containing the MLL-AF9 oncogene to create an ALL cell line that was then xenografted into the humanized NSGs. CAR T-cells were then created from humanized T-cells derived from the humanized mice, generating a humanized autologous CAR T-cell mouse model that represents the current therapeutic approach of most CAR T-cell products. However, the system did not recapitulate CRS, which was thought to be due to the lack of human cytokine transgene expression^159^.

A long-term goal of CAR therapy is to produce CAR T-cells *in vivo.* To test this in preclinical models, NSG mice were xenografted with the Raji^+^ cell line, human B-cell-depleted peripheral blood mononuclear cells (PBMCs), and lentivirus targeted to CD8^+^ T-cells containing CAR19 genes^160^. These experiments showed that Raji^+^ lymphoblastic cells and normal CD19^+^ cells can be eliminated via the administration of lentivirus containing CAR T genes *in vivo*, with elevation of cytokines similar to CRS in some animals^160^.

The use of CAR technology in solid tumors has had less clinical success so far and remains an area of active investigation. CAR T-cells targeting GD2 and L1-CAM have been generated for use in neuroblastoma with good results in mouse models but limited success in clinical trials^75^. Based on promising preclinical evidence in xenograft models, CAR NK T-cells (NKTs) are beginning to be tested in solid tumors including neuroblastoma^161^. Furthermore, NKTs enriched for CD62L expression via artificial antigen-presenting cell-mediated expansion have been used to produce CAR NKTs and showed that CD62L^+^ CAR NKTs have fivefold longer persistence in host mice than non-CD62L^–^ CAR NKTs, suggesting another promising avenue for study^162^. Numerous studies have explored methods for enhancing the efficacy of CAR T-cells in the solid TME. T-cell exhaustion and tumor PD-L1/2 expression have been postulated as limiting factors for the efficacy of CAR T-cells in the solid TME^163^. Enhancing CAR T-cell function with immunomodulators including the TGF-β inhibitor galunisertib, checkpoint inhibitors, and lenalidomide have been tried with promising preclinical results^163,164^. CAR T-cells targeting B7-H3 (CD276), a checkpoint molecule highly expressed on pediatric solid tumor and brain tumors, have been generated for use in preclinical orthotopic xenograft models of osteosarcoma, Ewing sarcoma, and medulloblastoma. Treatment with CAR T-cells targeting B7-H3 in preclinical models was promising, demonstrating tumor regression and improved survival in all three tumor types^165^.

### Checkpoint inhibitors

Checkpoint inhibitors represent another arm of immunotherapy with exceptional clinical activity in select malignancies including melanoma and HL yet no activity in other diseases. Checkpoint inhibitors function by increasing immune activation against a tumor; therefore, both an intact immune system and a TME are required. Checkpoint inhibitors have been studied in syngeneic murine models. These models have the advantage of intact immune systems but are limited by rapid murine tumor growth, absence of human targets, and lack of genetic complexity^7^. Humanized mice have therefore gained significant importance in the study of these agents.

Humanized models using NSG or RAG1 or 2^–/–^, IL2Rγ^null^ mice engrafted with human PBMCs and human-derived malignancies have been used to study checkpoint immunotherapy. One group xenografted humanized mice with human colorectal cancer or gastric cancer cells and treated them with nivolumab, a PD-1 inhibitor, and urelumab, a 4-1BB (CD137) agonist, alone and in combination, to evaluate their anti-tumor properties^65^. These studies demonstrated that mice treated with urelumab/nivolumab combination had increased T-cell IFN-γ expression, increased tumor CD8^+^ T-cell/T-regulatory cell ratios, and slowed colon cancer and gastric cancer tumor growth compared with untreated controls^65^. These studies suggest humanized mice may be relevant models for the study of checkpoint inhibitors in pediatric cancers. One major limitation of humanized mouse models in the study of checkpoint inhibitors is the xGVHD that frequently develops, with human T-cell infiltration into the liver, spleen, and lungs. xGVHD does limit the durations of studies but can be overcome with CD4 depletion^65^.

The immune PDX model provides another method for the study of checkpoint inhibitors. In this model, human tumors with associated tumor-infiltrating lymphocytes (TILs) are orthotopically placed in immunodeficient mice. Notably, these models can be used only as first passage models and only have a preserved immune system–tumor interaction for approximately 3–4 weeks post engraftment^7,166^.

Another model useful in the study of checkpoint inhibitors is the BALB/c RAG2^–/–^IL2Rγ^null^ mouse with transgenic expression of M-CSF, IL-3, GM-CSF, thrombopoietin, and SIRPɑ, for human immune system development, and are called MI(S)TRG. Humanized MI(S)TRG mice given human CD34^+^ cells were xenografted with human melanoma cell line Me290 and demonstrated human macrophage infiltration into xenografted melanoma, more closely recapitulating a human TME^53^. Given that the lack of an innate immune system has been a limitation of preclinical immunotherapeutic investigations, transgenic expression of human cytokines offers an important method for assessing immunotherapies with a more intact immune system.

While the above models have their limitations, they provide a closer look into human immune response to human malignancy. The use of these xenograft models with combination therapies (checkpoint inhibitors + conventional chemotherapy or checkpoint inhibitors + adoptive cell therapy) are likely to provide useful information about this expanding area of investigation in cancer immunotherapy.

## Conclusions

The increasing understanding of the influence of genetics, epigenetics, and proteomics on human cancer behavior, combined with the development of PDX biobanks, comprehensive molecular profiling, high-throughput drug screening, and xenograft models allow us to study a wider variety of cancers in a more predictive way than ever before. Persistent challenges include the influence of mouse stroma-infiltrating human tumors, xGVHD, difficulties recapitulating the human immune system and TME, genetic drift, and clonal evolution with serial passage of cancer cells. As precision medicine and immunotherapies have become more commonplace, accurate model systems remain of utmost importance.
